# Supplementary material for: Ouabain and Digoxin Activate the Proteasome and the Degradation of the ERα in Cells Modeling Primary and Metastatic Breast Cancer
Source: Cancers (Basel). 2020 Dec 19;12(12):3840. doi: 10.3390/cancers12123840 (PMC7766733; doi:10.3390/cancers12123840)
Supplement: Supplementary file 1 [file cancers-12-03840-s001.zip › supplement/Figures S1-S5.pptx]

## Slide 1
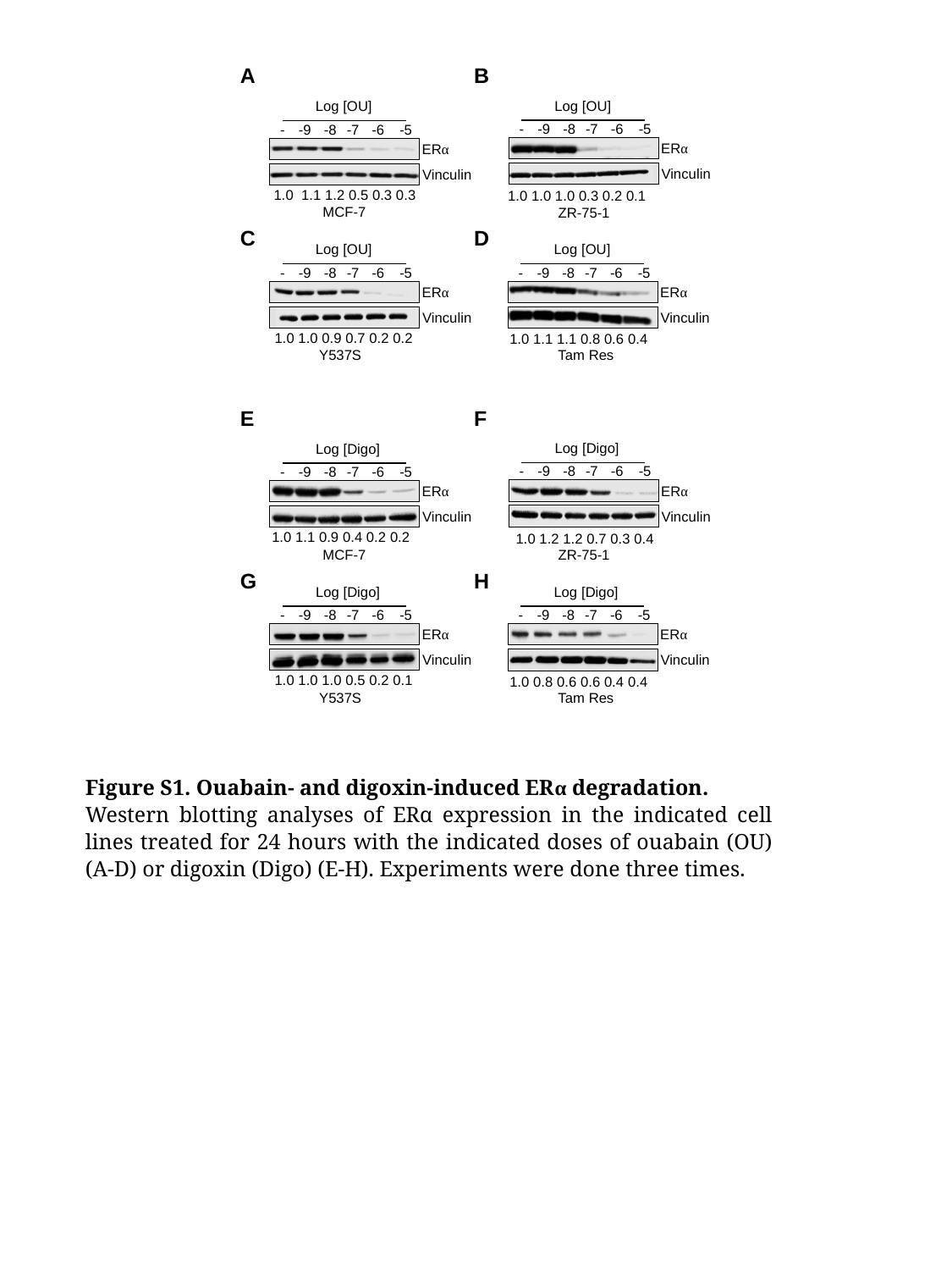

A
B
Log [OU]
-
-9
-8
-7
-6
-5
ERα
Vinculin
1.0 1.0 1.0 0.3 0.2 0.1
ZR-75-1
Log [OU]
-
-9
-8
-7
-6
-5
ERα
Vinculin
1.0 1.1 1.2 0.5 0.3 0.3
MCF-7
C
D
Log [OU]
-
-9
-8
-7
-6
-5
ERα
Vinculin
1.0 1.0 0.9 0.7 0.2 0.2
Y537S
Log [OU]
-
-9
-8
-7
-6
-5
ERα
Vinculin
1.0 1.1 1.1 0.8 0.6 0.4
Tam Res
E
F
Log [Digo]
-
-9
-8
-7
-6
-5
ERα
Vinculin
1.0 1.2 1.2 0.7 0.3 0.4
ZR-75-1
Log [Digo]
-
-9
-8
-7
-6
-5
ERα
Vinculin
1.0 1.1 0.9 0.4 0.2 0.2
MCF-7
G
H
Log [Digo]
-
-9
-8
-7
-6
-5
ERα
Vinculin
1.0 1.0 1.0 0.5 0.2 0.1
Y537S
Log [Digo]
-
-9
-8
-7
-6
-5
ERα
Vinculin
1.0 0.8 0.6 0.6 0.4 0.4
Tam Res
Figure S1. Ouabain- and digoxin-induced ERα degradation.
Western blotting analyses of ERα expression in the indicated cell lines treated for 24 hours with the indicated doses of ouabain (OU) (A-D) or digoxin (Digo) (E-H). Experiments were done three times.

## Slide 2
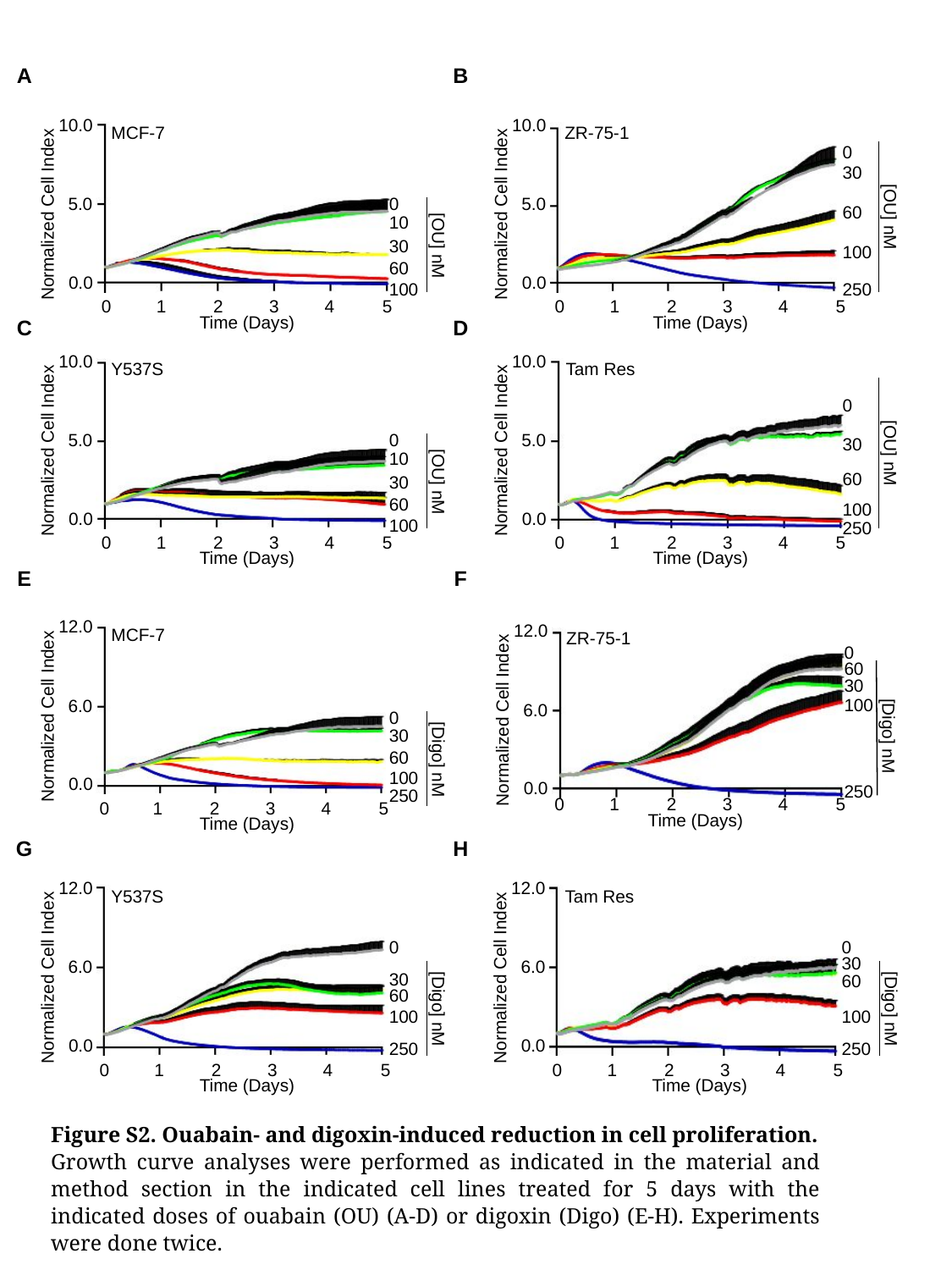

A
B
10.0
MCF-7
0
5.0
Normalized Cell Index
10
30
[OU] nM
60
0.0
100
0
1
2
3
4
5
Time (Days)
10.0
ZR-75-1
0
30
5.0
60
Normalized Cell Index
[OU] nM
100
0.0
250
0
1
2
3
4
5
Time (Days)
C
D
10.0
Y537S
0
5.0
Normalized Cell Index
10
30
[OU] nM
60
0.0
100
0
1
2
3
4
5
Time (Days)
10.0
Tam Res
0
5.0
30
Normalized Cell Index
[OU] nM
60
100
0.0
250
0
1
2
3
4
5
Time (Days)
E
F
12.0
MCF-7
6.0
Normalized Cell Index
0
30
60
[Digo] nM
100
0.0
250
0
1
2
3
4
5
Time (Days)
12.0
ZR-75-1
0
60
30
100
6.0
Normalized Cell Index
[Digo] nM
0.0
250
0
1
2
3
4
5
Time (Days)
G
H
12.0
Y537S
0
6.0
Normalized Cell Index
30
60
[Digo] nM
100
0.0
250
0
1
2
3
4
5
Time (Days)
12.0
Tam Res
0
30
6.0
Normalized Cell Index
60
[Digo] nM
100
0.0
250
0
1
2
3
4
5
Time (Days)
Figure S2. Ouabain- and digoxin-induced reduction in cell proliferation.
Growth curve analyses were performed as indicated in the material and method section in the indicated cell lines treated for 5 days with the indicated doses of ouabain (OU) (A-D) or digoxin (Digo) (E-H). Experiments were done twice.

## Slide 3
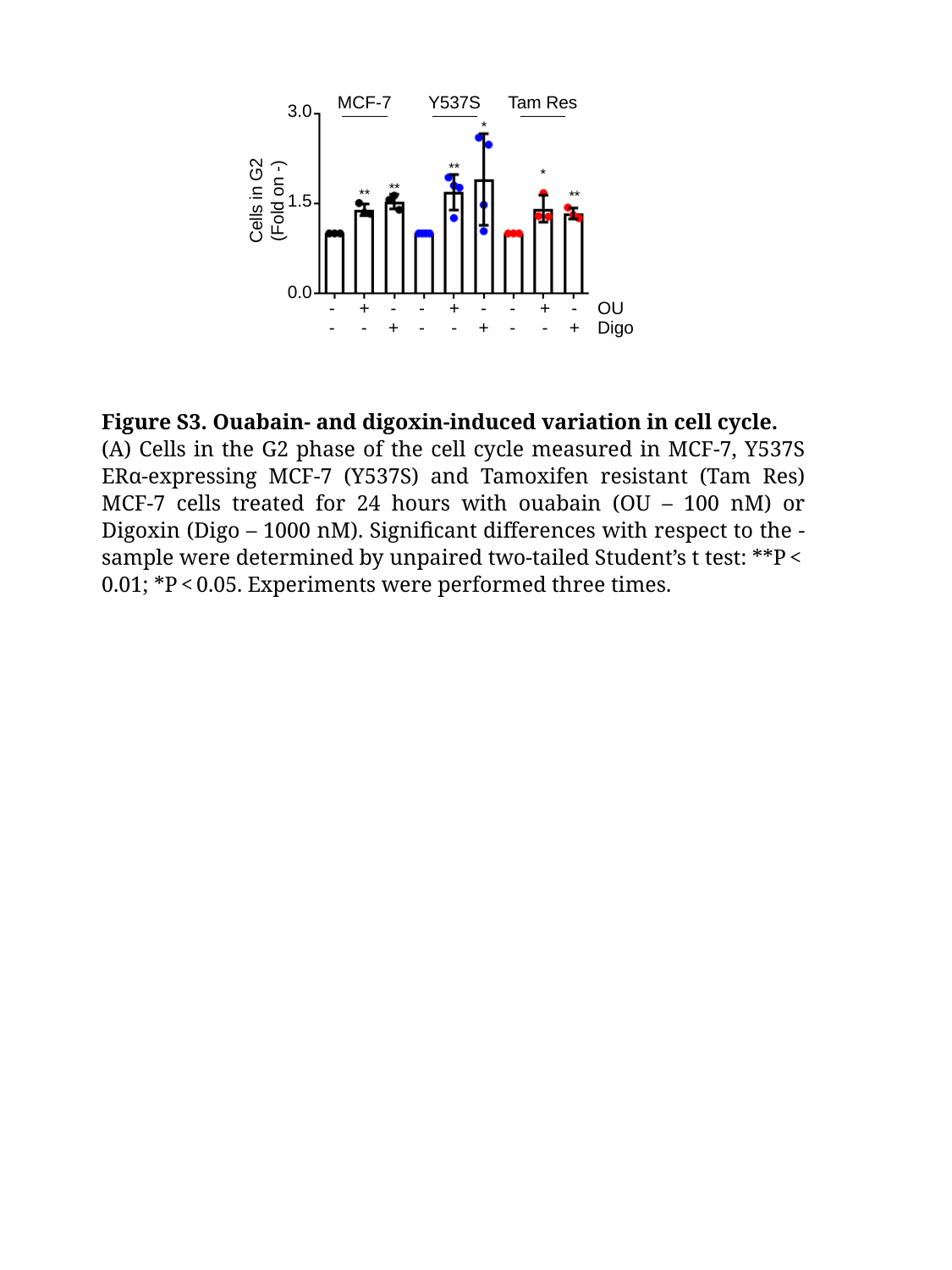

MCF-7
Y537S
Tam Res
3.0
Cells in G2
(Fold on -)
1.5
0.0
-
+
-
-
-
+
-
+
-
-
-
+
-
+
-
-
-
+
OU
Digo
*
**
*
**
**
**
Figure S3. Ouabain- and digoxin-induced variation in cell cycle.
(A) Cells in the G2 phase of the cell cycle measured in MCF-7, Y537S ERα-expressing MCF-7 (Y537S) and Tamoxifen resistant (Tam Res) MCF-7 cells treated for 24 hours with ouabain (OU – 100 nM) or Digoxin (Digo – 1000 nM). Significant differences with respect to the - sample were determined by unpaired two-tailed Student’s t test: **P < 0.01; *P < 0.05. Experiments were performed three times.

## Slide 4
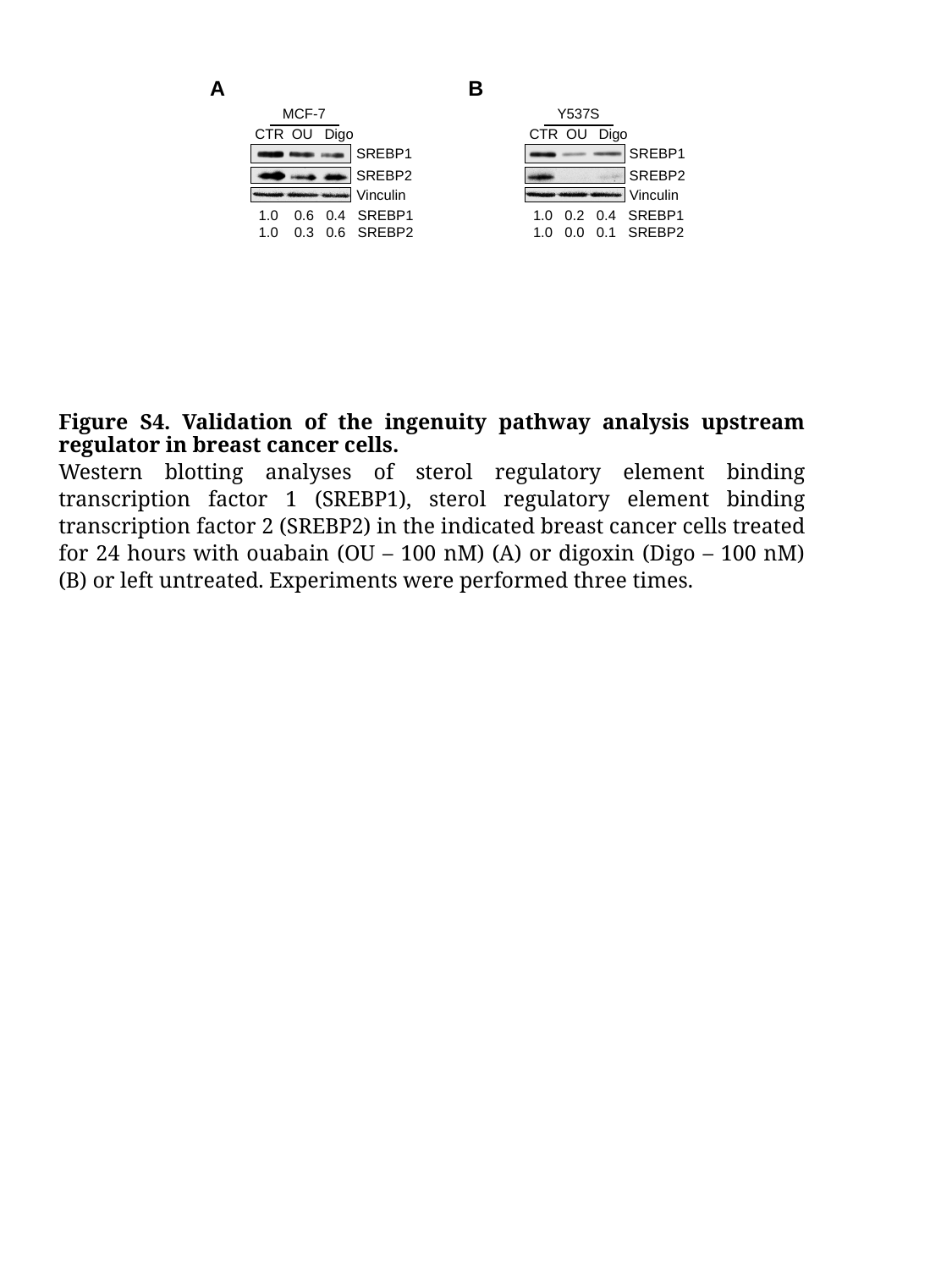

A
B
MCF-7
CTR
OU
Digo
SREBP1
SREBP2
Vinculin
1.0 0.6 0.4 SREBP1
1.0 0.3 0.6 SREBP2
Y537S
CTR
OU
Digo
SREBP1
SREBP2
Vinculin
1.0 0.2 0.4 SREBP1
1.0 0.0 0.1 SREBP2
Figure S4. Validation of the ingenuity pathway analysis upstream regulator in breast cancer cells.
Western blotting analyses of sterol regulatory element binding transcription factor 1 (SREBP1), sterol regulatory element binding transcription factor 2 (SREBP2) in the indicated breast cancer cells treated for 24 hours with ouabain (OU – 100 nM) (A) or digoxin (Digo – 100 nM) (B) or left untreated. Experiments were performed three times.

## Slide 5
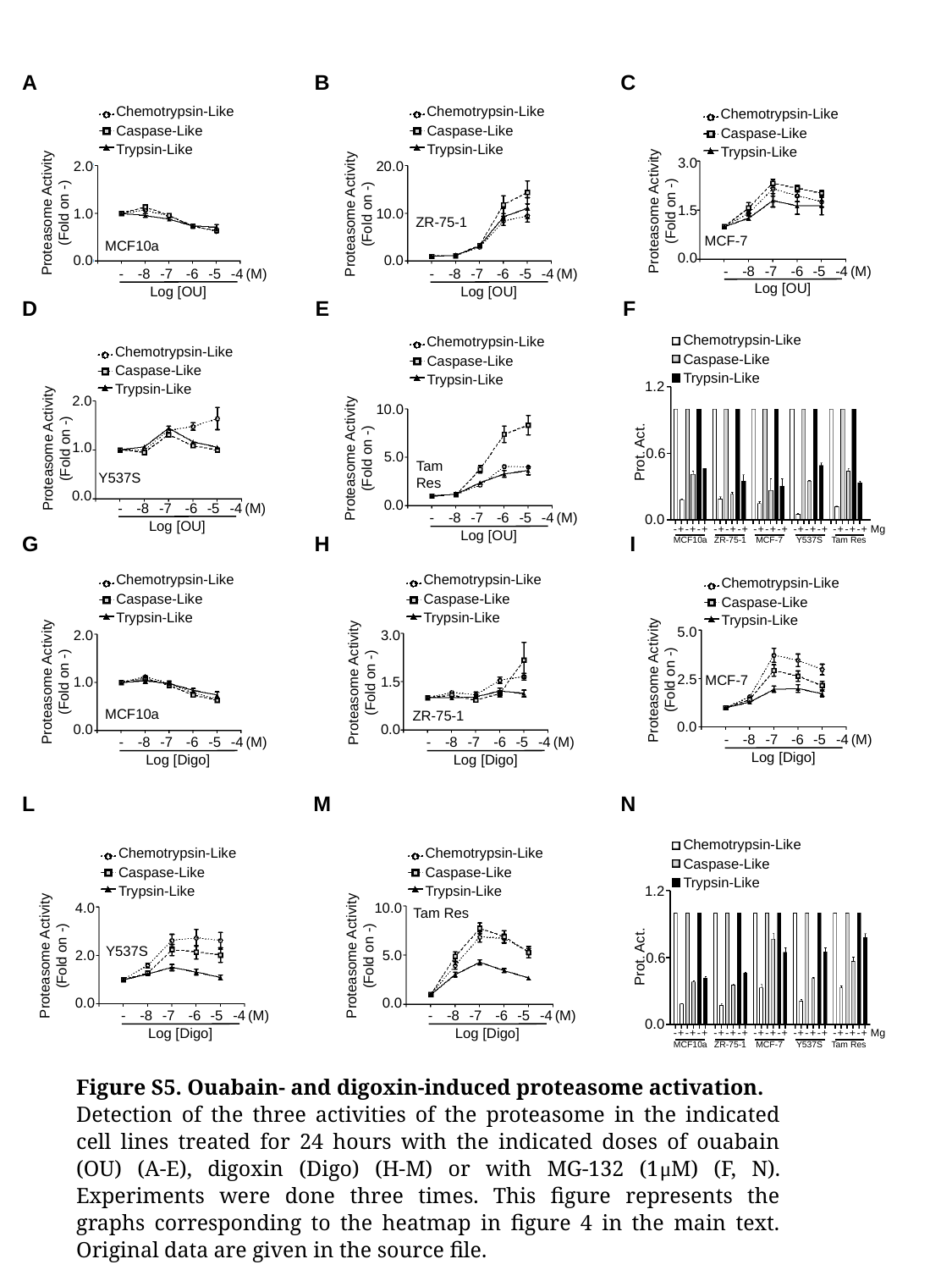

A
B
C
Chemotrypsin-Like
Caspase-Like
Trypsin-Like
2.0
Proteasome Activity
(Fold on -)
1.0
MCF10a
0.0
-
-8
-7
-6
-5
-4
(M)
Log [OU]
Chemotrypsin-Like
Caspase-Like
Trypsin-Like
20.0
Proteasome Activity
(Fold on -)
10.0
ZR-75-1
0.0
-
-8
-7
-6
-5
-4
(M)
Log [OU]
Chemotrypsin-Like
Caspase-Like
Trypsin-Like
3.0
Proteasome Activity
(Fold on -)
1.5
MCF-7
0.0
-
-8
-7
-6
-5
-4
(M)
Log [OU]
D
E
F
Chemotrypsin-Like
Caspase-Like
Trypsin-Like
1.2
Prot. Act.
0.6
0.0
-
+
-
+
-
+
MCF10a
-
+
-
+
-
+
ZR-75-1
-
+
-
+
-
+
MCF-7
-
+
-
+
-
+
Y537S
-
+
-
+
-
+
Tam Res
Mg
Chemotrypsin-Like
Caspase-Like
Trypsin-Like
10.0
Proteasome Activity
(Fold on -)
5.0
Tam
Res
0.0
-
-8
-7
-6
-5
-4
(M)
Log [OU]
Chemotrypsin-Like
Caspase-Like
Trypsin-Like
2.0
Proteasome Activity
(Fold on -)
1.0
Y537S
0.0
-
-8
-7
-6
-5
-4
(M)
Log [OU]
G
H
I
Chemotrypsin-Like
Caspase-Like
Trypsin-Like
2.0
Proteasome Activity
(Fold on -)
1.0
MCF10a
0.0
-
-8
-7
-6
-5
-4
(M)
Log [Digo]
Chemotrypsin-Like
Caspase-Like
Trypsin-Like
3.0
Proteasome Activity
(Fold on -)
1.5
ZR-75-1
0.0
-
-8
-7
-6
-5
-4
(M)
Log [Digo]
Chemotrypsin-Like
Caspase-Like
Trypsin-Like
5.0
Proteasome Activity
(Fold on -)
2.5
MCF-7
0.0
-
-8
-7
-6
-5
-4
(M)
Log [Digo]
L
M
N
Chemotrypsin-Like
Caspase-Like
Trypsin-Like
1.2
Prot. Act.
0.6
0.0
-
+
-
+
-
+
MCF10a
-
+
-
+
-
+
ZR-75-1
-
+
-
+
-
+
MCF-7
-
+
-
+
-
+
Y537S
-
+
-
+
-
+
Tam Res
Mg
Chemotrypsin-Like
Caspase-Like
Trypsin-Like
4.0
Proteasome Activity
(Fold on -)
Y537S
2.0
0.0
-
-8
-7
-6
-5
-4
(M)
Log [Digo]
Chemotrypsin-Like
Caspase-Like
Trypsin-Like
10.0
Tam Res
Proteasome Activity
(Fold on -)
5.0
0.0
-
-8
-7
-6
-5
-4
(M)
Log [Digo]
Figure S5. Ouabain- and digoxin-induced proteasome activation.
Detection of the three activities of the proteasome in the indicated cell lines treated for 24 hours with the indicated doses of ouabain (OU) (A-E), digoxin (Digo) (H-M) or with MG-132 (1μM) (F, N). Experiments were done three times. This figure represents the graphs corresponding to the heatmap in figure 4 in the main text. Original data are given in the source file.
